# A leaky umbrella has little value: evidence clearly indicates the serotonin system is implicated in depression

**DOI:** 10.1038/s41380-023-02095-y

**Published:** 2023-06-16

**Authors:** Sameer Jauhar, Danilo Arnone, David S. Baldwin, Michael Bloomfield, Michael Browning, Anthony J. Cleare, Phillip Corlett, J. F. William Deakin, David Erritzoe, Cynthia Fu, Paolo Fusar-Poli, Guy M. Goodwin, Joseph Hayes, Robert Howard, Oliver D. Howes, Mario F. Juruena, Raymond W. Lam, Stephen M. Lawrie, Hamish McAllister-Williams, Steven Marwaha, David Matuskey, Robert A. McCutcheon, David J. Nutt, Carmine Pariante, Toby Pillinger, Rajiv Radhakrishnan, James Rucker, Sudhakar Selvaraj, Paul Stokes, Rachel Upthegrove, Nefize Yalin, Lakshmi Yatham, Allan H. Young, Roland Zahn, Philip J. Cowen

**Affiliations:** 1 Centre for Affective Disorders, Psychological Medicine, IoPPN, King’s College, London, UK; 2 Department of Psychiatry and Behavioural Science, College of Medicine and Health Sciences, United Arab Emirates University, Al Ain, United Arab Emirates; 3 Clinical and Experimental Sciences, Faculty of Medicine, University of Southampton, Southampton, UK; 4 Mental Health Neuroscience, Division of Psychiatry, University College, London, UK; 5 Department of Psychiatry, University of Oxford, Oxford, United Kingdom; Oxford Health NHS Trust, Oxford, United Kingdom; 6 Department of Psychiatry, Yale School of Medicine, New Haven, CT 06519 USA; 7 Department of Psychiatry, University of Manchester, Manchester, UK; 8 Division of Brain Sciences, Dept of Medicine, Imperial College, London, UK; 9 Department of Psychological Sciences, School of Psychology, University of East London, London, UK; 10 Department of Psychosis Studies, IoPPN, King’s College, London, UK; 11 H Lundbeck A/s, Iveco House, Watford, WD17 1ET UK; 12 Institute of Clinical Sciences (ICS), Faculty of Medicine, Imperial College London, Du Cane Road, London, UK; 13 Department of Psychiatry, University of British Columbia, Vancouver, BC Canada; 14 Division of Psychiatry, University of Edinburgh, Edinburgh, UK; 15 Faculty of Medical Sciences, University of Newcastle, Newcastle upon Tyne, UK; 16 Translational and Clinical Research Institute, Newcastle University, Newcastle upon Tyne, UK; 17 Institute for Mental Health, University of Birmingham, Birmingham, UK; 18 Departments of Radiology and Biomedical Sciences, Psychiatry, and Neurology, Yale University, New Haven, CT USA; 19 Yale Institute for Global Health, New Haven, CT USA; 20 Louis Faillace Department of Psychiatry and Behavioral Science, McGovern Medical School, University of Texas Health Science Center, Houston, TX USA; 21 Present Address: Intra-Cellular Therapies, Inc, New York, NY 10016 USA

**Keywords:** Neuroscience, Molecular biology

## Abstract

A recent “umbrella” review examined various biomarkers relating to the serotonin system, and concluded there was no consistent evidence implicating serotonin in the pathophysiology of depression. We present reasons for why this conclusion is overstated, including methodological weaknesses in the review process, selective reporting of data, over-simplification, and errors in the interpretation of neuropsychopharmacological findings. We use the examples of tryptophan depletion and serotonergic molecular imaging, the two research areas most relevant to the investigation of serotonin, to illustrate this.

Moncrieff et al. [1] aimed to synthesise and evaluate evidence on serotonin (5-hydroxytryptamine, 5-HT) in the context of the monoamine hypothesis of depression, into an umbrella review. They concluded, “there is no convincing evidence that depression is associated with, or caused by, lower serotonin concentrations or activity.” The review included investigations of 5-HT and its metabolite 5-hydroxyindoleacetic acid (5-HIAA) in “body fluids”, 5HT_1A_ receptor and serotonin transporter protein (SERT) availability in imaging and post-mortem studies, investigations of SERT gene polymorphisms, interactions between SERT and stress in depression, and effects of tryptophan depletion on mood. Given the complexity of the serotonergic system and importance of the subject, great care and transparency are essential when reviewing, synthesising and interpreting such data. Clear guidance is available for conducting umbrella reviews, cited in the current review [2].

Putting aside concerns about the antiquated concept of single-gene polymorphisms and the current view of polygenetic architecture of Major Depressive Disorder (MDD), and discussion of antidepressant efficacy in a review that did not present any such data, we highlight substantial methodological weaknesses that make interpretation of the current review challenging.

## Methodological weaknesses in the review

The evidence synthesis approach adopted [1] has several inherent methodological weaknesses. Whilst umbrella reviews typically split their study-unit into previous meta-analyses/systematic reviews of primary studies *vs*. umbrella reviews of meta-analyses/systematic reviews (also termed “meta-umbrella” reviews) [3], the authors summarised these different study units together, appearing to include some primary studies at the expense of others (see below, exclusion of Yatham et al. [4], for example). This makes comparative interpretation of effect sizes potentially unreliable. This is complicated by heterogeneity of the summarised measures (genetic, neuroimaging, biochemical) and methodological choices, such as not synthesising ‘results of individual meta-analyses because they included overlapping studies’ [1]. This is in contrast to umbrella reviews that have selected non-overlapping primary studies from overlapping meta-analyses [3]. Validated criteria are established to classify levels of evidence in umbrella reviews of biomarkers, to avoid selective reporting and post-hoc manipulation [3]. The authors did not use these: interpretation of evidence was undertaken with a mixture of metrics, including a “modified” version of GRADE quality assessment, introduced in a *post-hoc* protocol amendment. GRADE initially categorises observational studies as “low”, and these are downgraded or upgraded based on pre-specified criteria. The authors rate certainty of evidence determined “by consensus of at least two authors and a direction of effect indicated” [1]. The authors cite an original meta-analysis [5] to justify their choice, but there is little similarity between the two studies. Criteria in the current review were unified statistical analysis of original data, confounding by antidepressant use adequately excluded where effect found, outcomes of interest pre-specified, consistent results, little likelihood of publication bias (in funnel plots or tests) and large sample (*n* > 500, non-genetic studies, >10,000 for genetic studies). It is unclear why confounding effects of antidepressants would not apply to all studies, i.e. not just where positive outcomes were seen: antidepressants are as likely to confound studies with negative results (a point acknowledged by the authors in the review).

## A depleted account of tryptophan depletion

These methodological weaknesses are manifested in the consideration of tryptophan depletion studies. The authors include a meta-analysis, systematic review and 10 recent studies involving healthy volunteers, but missed a clinical and molecular imaging study, which showed an effect in people with MDD [4]. The authors state, “studies involving people diagnosed with depression showed slightly greater mood reduction following tryptophan depletion…but most participants were taking antidepressants and participant numbers were small.” However, in the meta-analysis they cite [6], the effect size for the effects of tryptophan depletion on mood in depressed people not taking antidepressants, from 8 samples, was large (Hedge’s g = −1.9 (95% CIs −3.02 to −0.78). Admittedly, a number of these studies came from the same group, and confidence intervals were wide (removal of a potential outlier decreased Hedge’s g to −1.06 (95% CIs −1.83 to −0.29)). Notwithstanding this, omission of these data in the current review is questionable. A more accurate interpretation is that tryptophan depletion studies suggest a role for 5-HT in people vulnerable to depression and in those remitted on SSRI treatment. In contrast, by citing a series of individual negative studies in healthy participants, the authors give the impression tryptophan depletion has no effect.

The authors also omit several studies included in 2 meta-analyses [7, 8] of circulating tryptophan concentrations, which directly influence central serotonin synthesis. This is far more relevant to CNS serotonin function than serotonin and 5-HIAA levels in body fluids, which are covered in the review. L-tryptophan plasma concentrations show, after adjusting for publication bias, significant decrease in people with MDD (Hedge’s g = −0.45, 95% CIs, −0.66 to −0.23), with a large effect size of g = −0.84 (95% CIs −1.27 to −0.4) in unmedicated people [7]. Furthermore, using the authors’ bespoke criteria for certainty, this would presumably score quite well given that it fulfils most criteria, except pre-specification of outcome measures. The authors were aware of this meta-analysis as they state they excluded it from the section on serotonin in body fluids.

## Simplistic (mis)interpretation of molecular imaging evidence

The authors misrepresent and misinterpret molecular imaging data regarding both 5-HT_1A_ receptor and serotonin transporter protein (SERT) binding. They cite a meta-analysis involving 10 5-HT_1A_ receptor studies [9], most of which used BP_ND_, an outcome measure of receptor availability not requiring arterial input, which assumes there exists a “reference region” with no specific binding in MDD and controls; [10] moreover, 5 studies included people with bipolar disorder or post-partum depression. The authors also make the simplistic interpretation that reduced binding would suggest increased synaptic 5-HT. However, decreased binding can be the result of several factors, including decreased receptor density or affinity. The authors state, “5HT_1A_ receptors, known as autoreceptors”, mistakenly assuming 5HT_1A_ receptors are exclusively pre-synaptic autoreceptors. Most are post-synaptic 5-HT_1A_ heteroreceptors. Diminished availability of post-synaptic 5-HT_1A_ receptors in unmedicated depression would be consistent with lowered 5-HT neurotransmission. Whilst most studies report lower 5-HT_1A_ binding, higher binding is seen if a more sophisticated outcome measure with arterial input is used, and lower binding noted when the reference region approach taken in most studies is used. This is a replicated finding in people free of antidepressants for a period of years [10]. None of this complexity is acknowledged in the review.

## An uncertain approach to SERT binding

The authors conclude, in appraising three reviews of SERT binding, that the “areas in which effects were detected were not consistent”. However, findings in a number of brain regions *are* consistent, with SERT reductions reported in people with MDD in all reviews. This is seen in Table 1 in the current review. A review they describe as a meta-analysis (and included twice, for 5HT_!1A_ and SERT studies) appears only to pool values, and all reviews analysed different brain regions. This question would have been legitimately answered by a conventional umbrella review, where included studies are extracted and meta-analysed. Furthermore, the modified GRADE scoring appears unclear- in one review [11], antidepressant use was an exclusion in context of a positive outcome, though scored as 0. Publication bias is reported as a test statistic, though again scored 0. The authors make another over-simplification, assuming lower SERT binding is associated with higher synaptic 5-HT. The reference cited presents other models, including lower SERT binding associated with *lower* 5-HT [12]. In-vivo studies have failed to show a relationship between SERT and endogenous serotonin [11, 13], and the relationship between decreased SERT and MDD could be explained through mechanisms such as decreased SERT expression in neurons and/or decreased density of serotonergic neurons [11]. The authors’ explanation is that reduced SERT in meta-analyses is due to prior antidepressant treatment but appear unaware of reductions reported in drug-naïve populations [14]. They also appear to have missed the fact that 149 of 364 people in one of the cited meta-analyses were drug naïve [15], and therefore their conclusion that these meta-analytic findings were due to antidepressants is difficult to follow.

When commenting on the molecular imaging literature and its interpretation, it is worth noting individual studies, including a PET study which suggested decreased serotonin synthesis in medication-free people with MDD compared to controls [16], and a recent [^11^C]Cimbi-36 PET study of serotonin release with amphetamine challenge [17], which suggested reduced serotonin release capacity in 17 antidepressant-free people with a major depressive episode, compared to controls. Whilst the latter study was not available for the original researchers to include, it emphasises that curtailing research in this area is premature.

Furthermore, almost all effective antidepressants have an effect on the 5-HT system (including ketamine, in pre-clinical models [18], and 5-HT agonism remains one of the main mechanisms for the antidepressant qualities of psychedelics.

## Conclusion

To summarise, the methodology is inconsistent with an umbrella review, with substantial bias created by the authors’ chosen quality criteria, selective reporting, and interpretation of results. There is an underappreciation of the complexities of neuroscience and neuropsychopharmacology, and it is therefore impossible for the reader to draw valid or reliable conclusions.

A more accurate, constructive conclusion would be that acute tryptophan depletion and decreased plasma tryptophan in depression indicate a role for 5-HT in those vulnerable to or suffering from depression, and that molecular imaging suggests the system is perturbed. The proven efficacy of SSRIs in a proportion of people with depression lends credibility to this position.
